# Comparative Evaluation of Intestinal Absorption and Functional Value of Iron Dietary Supplements and Drug with Different Delivery Systems

**DOI:** 10.3390/molecules25245989

**Published:** 2020-12-17

**Authors:** Paolo Pastore, Marco Roverso, Erik Tedesco, Marta Micheletto, Etienne Mantovan, Michela Zanella, Federico Benetti

**Affiliations:** 1 Department of Chemical Sciences, University of Padova, Via Marzolo 1, 35131 Padova, Italy; paolo.pastore@unipd.it (P.P.); marco.roverso@unipd.it (M.R.); 2 ECSIN—European Center for the Sustainable Impact of Nanotechnology, ECAMRICERT SRL, Corso Stati Uniti 4, 35127 Padova, Italy; e.tedesco@ecamricert.com (E.T.); m.micheletto@ecamricert.com (M.M.); e.mantovan@ecamricert.com (E.M.); m.zanella@ecamricert.com (M.Z.)

**Keywords:** iron, dietary supplements, intestinal absorption, delivery systems, ferritin

## Abstract

Iron is a fundament micronutrient, whose homeostasis is strictly regulated. Iron deficiency anemia is among the most widespread nutritional deficiencies and its therapy, based on dietary supplement and drugs, may lead to severe side effects. With the aim of improving iron bioavailability while reducing iron oral therapy side effects, novel dietary supplements based on innovative technologies—microencapsulation, liposomes, sucrosomes—have been produced and marketed. In the present work, six iron dietary supplements for different therapeutic targets were compared in terms of bioaccessibility, bioavailability, and safety by using an integrated in vitro approach. For general-purpose iron supplements, ME + VitC (microencapsulated) showed a fast, burst intestinal iron absorption kinetic, which maintained iron bioavailability and ferritin expression constant over time. SS + VitC (sucrosomes), on the other side, showed a slower, time-dependent iron absorption and ferritin expression trend. ME + Folate (microencapsulated) showed a behavior similar to that of ME + VitC, albeit with a lower bioavailability. Among pediatric iron supplements, a time-dependent bioavailability increase was observed for LS (liposome), while PIC (polydextrose-iron complex) bioavailability is severely limited by its poor bioaccessibility. Finally, except for SS + VitC, no adverse effects on intestinal mucosa vitality and barrier integrity were observed. Considering obtained results and the different therapeutic targets, microencapsulation-based formulations are endowed with better performance compared to the other formulations. Furthermore, performances of microencapsulated products were obtained with a lower iron daily dose, limiting the potential onset of side effects.

## 1. Introduction

Iron (Fe) is one of the most relevant trace elements, since it plays an important role in many physiological processes such as oxygen metabolism, oxygen uptake, energy production, and hematopoiesis, and human well-being. In healthy adults, about 0.5–2 mg of iron is lost daily [1] due to blood loss and iron-containing epithelial cell exfoliation from gastrointestinal and urinary tracts, skin, and hair. To preserve its homeostasis, iron should be introduced from dietary sources and absorbed at the intestinal level.

Due to its ability to induce reactive oxygen species (ROS) and oxidative damages under non-physiological and pathological conditions [2,3], iron metabolism is strictly regulated at absorption, transport, storage, and excretion levels [4]. Nevertheless, about 12% of the world’s population is anemic due to iron deficiency [5,6]. The increase in iron demand could be linked to low amounts in human body iron stores (e.g., body growth, pregnancy, and lactation) [7,8,9], limited external supply and absorption (e.g., poor intake, inappropriate diet, malabsorption, genetic diseases), [10,11,12] and/or increased iron loss (e.g., bleeding trauma, menses, and multiparity) [13,14,15]. To avoid iron deficiency, iron supplementation via dietary supplements would be recommended [16]. Iron supplementation through the oral route is more patient compliant compared to intravenous iron supplementation and red blood cell transfusion. Commonly used oral iron salts are poorly absorbed [17], with unabsorbed iron leading to several gastrointestinal side effects [18,19,20]. These have led to a decrease in iron-based supplements compliance, significantly undermining the long-term efficacy of oral iron supplements. A new generation of iron dietary supplements with ad hoc iron delivery systems, such as microencapsulated iron [21], liposomes [22,23], and sucrosomes [24] have been produced. Such technologies ensure for iron protection during the digestive process, limiting the release of unabsorbed iron, while improving iron bioavailability by exploiting new, non-iron-dependent intestinal absorption routes (i.e., transcytotic and paracellular routes) [25].

In the present work, six different iron delivery technologies developed for specific therapeutic targets are compared in terms of bioaccessibility, bioavailability, iron functional value, and safety. For general-purpose iron supplementation, the two ferric iron-based dietary supplements—microencapsulated (ME, with vitamin C) and sucrosome (SS, with vitamin C)—are compared. The microencapsulated dietary supplement is based on ferric iron (Fe^3+^) micronization, while sucrosome delivers ferric iron with a sucrester matrix (i.e., sucrosomes). Liposomes (LS) and polydextrose-iron complex (PIC) are usually produced and marketed for pediatric therapeutic purposes. While LS delivers ferrous iron (Fe^2+^), PIC transports ferric iron conjugated to a polydextrose-based matrix. Concerning obstetrics and gynecological therapeutic targets, microencapsulated (ME with folate) and iron-succinylated proteins complex (ISPC) formulations are investigated in the present study. The ME formulation contains microencapsulated ferric iron, while ISPC transports ferric iron in a succinylated proteins complex.

To our knowledge, this is the first scientific work that compares different delivery systems and technologies in their ability to improve iron intestinal absorption. Considering our results, iron bioaccessibility, bioavailability, and intestinal absorption mechanisms are significantly affected by the applied delivery technology. Among tested delivery systems, microencapsulation and liposome showed the best performances.

## 2. Results and Discussion

### 2.1. Fe Titer of Tested Formulations

The iron (Fe) content in tested formulations was analyzed, and the measured values were compared with nominal iron concentrations. The percentage of recovery was calculated as the ratio between measured and nominal Fe values. As reported in Table 1, measured iron contents in the formulations correspond to the declared amounts.

### 2.2. Bioaccessibility of the Fe-Based Formulations

Iron absorption from dietary supplements in iron deficient subject is usually low, peaking at 28% when consumed without food [17]. One of the factors potentially limiting iron absorption from dietary supplements is their ability to release iron during the digestive process, making it available for absorption at the intestinal level. The amount of active principle (i.e., Fe) release from its matrix (i.e., iron supplements) during the digestive process is defined as bioaccessibility.

**Figure 1:**
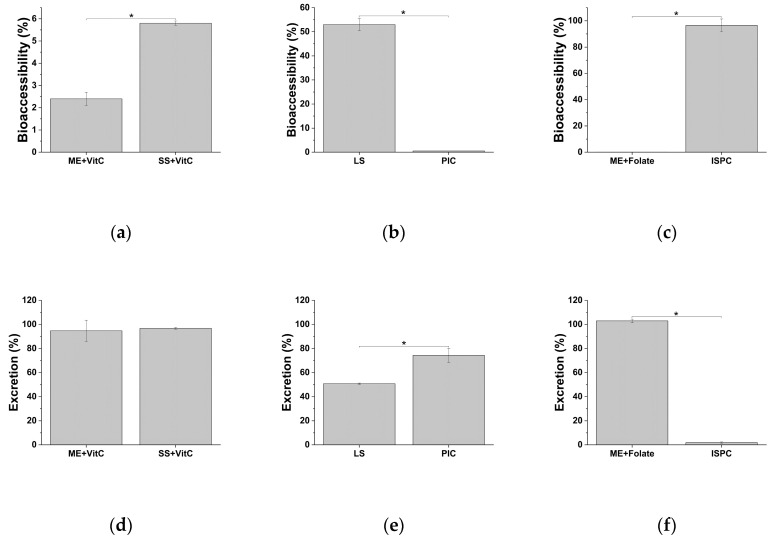
Iron bioaccessibility and excretion of general-purpose (**a**,**d**), pediatric (**b**,**e**), and obstetrics and gynecology (**c**,**f**) iron supplements. ME: Microencapsulation; SS: Sucrosome; LS: Liposome; PIC: Polydextrose–iron complex; ISPC: Iron-succinylated proteins complex. * *p* < 0.05.

To determine iron bioaccessibility of the formulations, daily doses were exposed to in vitro digestive process and Fe amount in digests (i.e., complete) and their fractions—supernatant (i.e., bioaccessible fraction) and pellet (i.e., excreted fraction)—were measured by ICP-MS (Inductively Coupled Plasma Mass Spectrometry). Among general-purpose iron supplements, ME + VitC shows significantly lower bioaccessibility compared to SS + VitC (Figure 1a and Table 2). Both formulations present limited bioaccessibility, as highlighted by the high iron content in the excreted fractions (Figure 1d and Table 2). The pediatric formulation LS is endowed with a better bioaccessibility than PIC (Figure 1b,e and Table 2). Obstetrics and gynecology iron supplements show the microencapsulated iron (ME + Folate) mainly excreted, while the iron bound to ISPC completely released from its matrix and became bioaccessible (Figure 1c,f and Table 2).

### 2.3. Functional Value of Iron in Tested Dietary Supplements after In Vitro Digestion

**Figure 2:**
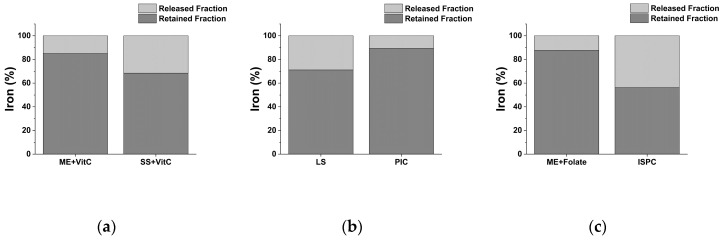
Functional iron expressed as retained and released fractions of general-purpose (**a**), pediatric (**b**), and obstetrics and gynecology (**c**) iron supplements after in vitro digestion and dialysis. ME: Microencapsulation; SS: Sucrosome; LS: Liposome; PIC: Polydextrose-iron complex; ISPC: Iron-succinylated proteins complex.

The aim of iron supplements is to prevent and/or mitigate anemia-correlated symptoms by increasing the amount of available and stored iron in the organism. While Fe absorption at the intestinal level can exploit different routes, the transcytosis pathway represents the most effective in rapidly increasing bioavailable iron. Conversely to the ionic form that is efficiently bound and stored by ferritin, transcytosis directly releases iron into the bloodstream. For these reasons, DLS was performed on digests to evaluate delivery system stability during digestive process. After exposure to digestion, delivery systems retain their particulate structure as showed in Table 3 and Figure S1. They are able to withstand the harsh biochemical conditions that are typical of the digestive process. Moreover, iron retained by delivery systems during the digestive process was assessed. After dialysis, released and retained iron fractions were measured by ICP-MS, and data are reported in Table 4. The formulations have significant differences in the ability to retain iron while avoiding its release (Table 4 and Figure 2). Among general-purpose formulations, SS + VitC presents the highest tendency to release iron, with the ME + VitC is more efficient in retaining it (Table 4 and Figure 2a). A similar behavior is observed for ME + Folate and ISPC, with the former being endowed with a higher iron retention capacity (Table 4 and Figure 2c). The PIC formulation is more effective in retaining iron into the delivery system compared to LS (Table 4 and Figure 2b).

### 2.4. Iron Intestinal Absorption

Once released from its matrix, iron needs to be effectively absorbed at the intestinal level to replenish iron storages and maintain iron homeostasis. The first absorption barrier encountered by iron in its course through the gastrointestinal tract is the duodenal enterocyte apical membrane that internalizes iron by different routes such as the divalent metal transporter (DMT-1) [26]. Innovative delivery systems can use transcytotic and paracellular routes for transporting iron into the human body. Considering that tested formulations are microencapsulated (ME + VitC and ME + Folate), liposome (LS), and sucrosome (SS + VitC) forms, iron intestinal absorption experiments were performed using a co-culture model made of human follicle associated intestinal epithelium, comprising mature enterocytes (Caco-2 cells) and microfold cells (M cells). This model allows for the investigation of iron absorption via DMT-1, transcytosis, and paracellular routes.

To evaluate their intestinal absorption, in vitro intestinal epithelia were exposed to bioaccessible fractions for 1 and 3 h. Intestinal iron absorption was evaluated considering both intracellular and basolateral contents as well as intracellular ferritin levels. Ferritin is used as an indicator of intracellular Fe content because it is involved in the micronutrient storage process, and its expression depends on intracellular Fe levels.

**Figure 3:**
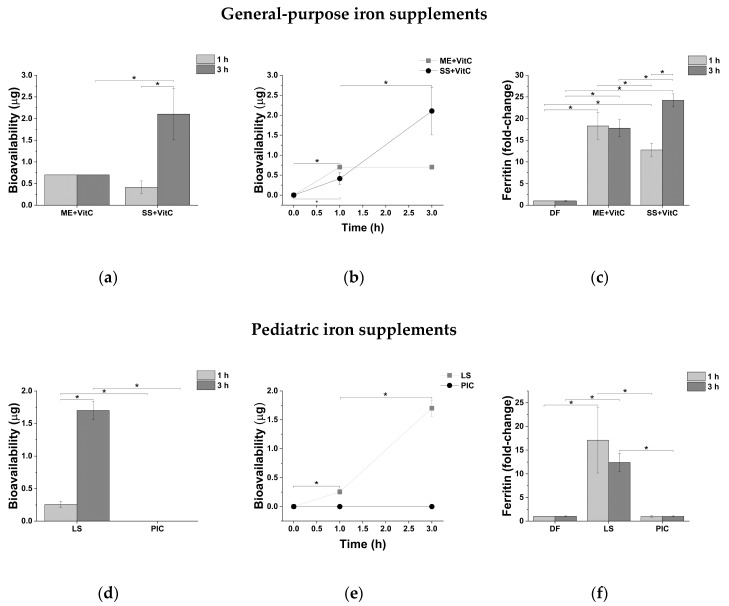

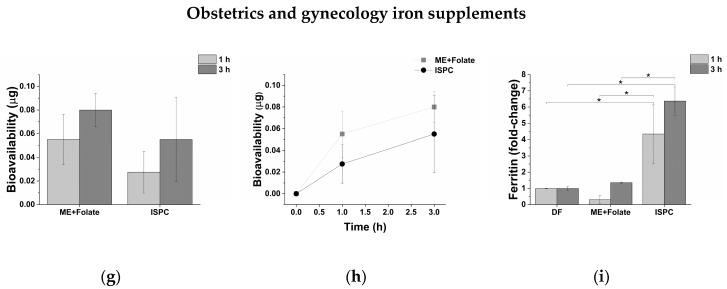
Iron intestinal bioavailability as amount (**a**,**d**,**g**), iron absorption kinetic (**b**,**e**,**h**), and ferritin expression (**c**,**f**,**i**) following intestinal epithelium exposure to general-purpose, pediatric and obstetrics, and gynecology iron supplements for 1 and 3 h. ME: Microencapsulation; SS: Sucrosome; LS: Liposome; PIC: Polydextrose-iron complex; ISPC: Iron-succinylated proteins complex; DF: digestive fluids. * *p* < 0.05.

Regarding general-purpose iron supplements, microencapsulate formulation (ME + VitC) shows a significantly higher intestinal absorption compared to SS + VitC after 1 h exposure (Figure 3a and Table 5). After 3 h exposure, the absorption of SS + VitC increases significantly, while no changes in ME + VitC absorption are observed (Figure 3a and Table 5). This indicates that ME + VitC is endowed with a fast absorption kinetic since peak absorption is reached following 1 h exposure. Conversely, SS + VitC reaches peak absorption later (3 h) showing a slower absorption kinetic (Figure 3b). The same trend is confirmed by ferritin analysis (Figure 3c and Table 5). As expected from its limited ionic iron release, the ferritin level of ME + VitC-treated intestinal epithelia remains unchanged over time, while it increases from 1 to 3 h following treatment with SS + VitC (Figure 3c and Table 5), which is endowed with a higher release of ionic iron than ME + VitC. Our results are in line with those available in the literature, which indicates that considering the same amount of iron, sucrosomes increases intestinal ferritin expression more than microencapsulated ferric iron [27,28]. This could depend on different absorption routes. Iron absorbed through transcytotic and paracellular routes should not affect ferritin expression, since it is not released in ionic form in the cell cytoplasm.

The comparison between iron supplements for pediatric purpose, LS and PIC, may support this hypothesis. Despite a time-dependent increase in intestinal Fe absorption (Figure 3d and Table 5) following treatment with LS, no significant increase in ferritin levels over time is observed (Figure 3f and Table 5). As expected from its poor bioaccessibility, no significant iron absorption and ferritin expression induction are observed for PIC at both considered time points (Figure 3d,f, Table 5). Among pediatric iron supplements, the microencapsulated formulation is endowed with better performance from the intestinal iron absorption point of view.

Despite their remarkable differences in bioaccessibility (about 96% and 0.1% for ISPC and ME + Folate respectively), no significant differences are observed at both time points between the bioavailability of obstetrics and gynecology iron supplements (Figure 3g and Table 5). For both formulations, a time-dependent increase in intestinal iron absorption is observed (Figure 3g,h, Table 5). Microencapsulated formulation ME + Folate does not induce an increase in ferritin expression over time, although there is a slight significant increase in iron bioavailability (Figure 3i and Table 5). This is in line with its ability to effectively retain iron without releasing it during digestion. On the contrary, ISPC induces an increase of ferritin expression in a time-dependent manner (Figure 3i and Table 5), which is probably due to the release of iron at the intestinal level (Table 2).

Considering microencapsulated formulations, the higher bioavailability of ME + VitC than ME + Folate could be explained by its larger bioaccessibility and the presence of ascorbic acid (vitamin C) that improves intestinal iron absorption [29,30]. Ferric acid (Fe^3+^), released from microencapsulated formulations, could be reduced to ferrous form (Fe^2+^) and readily absorbed at the intestinal level.

### 2.5. Impact of Fe-Based Formulations on Intestinal Epithelium

**Figure 4:**
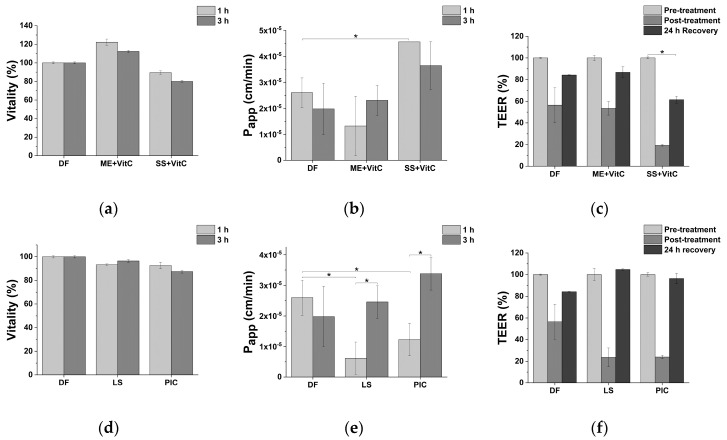

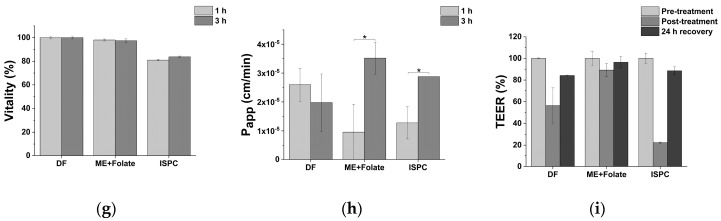
Impact of iron supplements on intestinal epithelium in vitro model vitality ((**a**): general-purpose iron supplements; (**d**): pediatric iron supplements; (**g**): obstetrics and gynecology iron supplements), apparent permeability (P_app_) (**b**): general-purpose iron supplements; (**e**): pediatric iron supplements; (**h**): obstetrics and gynecology iron supplements) and trans-epithelial electrical resistance (TEER) ((**c**): general-purpose iron supplements; (**f**): pediatric iron supplements; (**i**): obstetrics and gynecology iron supplements) following 1 and 3 h exposure. ME: Microencapsulation; SS: Sucrosome; LS: Liposome; PIC: Polydextrose-iron complex; ISPC: Iron-succinylated proteins complex; DF: Digestive fluids. * *p* < 0.05.

Iron supplementation may place a significant burden on gastrointestinal wellbeing. Due to limited iron absorption, supplemented iron could often cause gastric irritation, nausea, epigastric discomfort, and constipation. Free luminal iron likely results in enhanced catalytic activity and the production of reactive oxygen species within the intestine, leading to intestinal oxidative damages, irritation, inflammation, and intestinal dysfunction [31]. As such, iron dietary supplements to be effectively marketed need to be effective and safe. In this regard, the impact of tested iron supplements on intestinal epithelium vitality and barrier integrity was evaluated. As indicated in Figure 4, no significant alterations of intestinal epithelium vitality are observed after 1 h and 3 h treatment with general-purpose iron supplements (Figure 4a), pediatric iron supplements (Figure 4d), and obstetrics and gynecology iron supplements (Figure 4g). SS + VitC significantly increases intestinal epithelium permeability, as indicated by the increase in apparent permeability (Figure 4b) and the decrease in trans-epithelial electrical resistance (Figure 4c and Figure S2). Considering the available literature and the composition of sucrosomes, an increase in intestinal epithelium permeability is expected. Sucrester, a component of sucrosomes, is a surfactant, and it has been recently shown to behave as an absorption enhancer that facilitates sucrosomes absorption through transcytotic and paracellular pathways [32,33]. However, enhanced intestinal barrier permeability is stationary and not reversible. While a transient increase in intestinal epithelium could be instrumental in enhancing iron absorption, a prolonged exposure to absorption enhancer may increase the risk of intestinal infection due to the permeation of microorganisms from the microbiota. No significant effects on intestinal epithelium permeability are observed for the other tested iron supplements, for which only transient and reversible alterations are observed.

## 3. Materials and Methods

### 3.1. Materials

Caco-2 human epithelial colorectal adenocarcinoma cells (ATCC^®^ HTB-37™) and human Burkitt’s lymphoma derived Raji cells (ATCC^®^ CCL-86™) were purchased from ATCC (Manassas, VA, USA). High glucose Dulbecco’s Modified Eagle Medium (DMEM), Hanks’ Balanced Salt Saline (HBSS), non-essential amino acids (NEAA), L-glutamine, Penicillin-Streptomycin mix, reagents for simulant digestive fluids preparation, Lucifer Yellow (LY), and Human Ferritin ELISA Kit were purchased from Sigma-Aldrich (St Louis, MO, USA). Foetal bovine serum (FBS) was purchased from Euroclone (Milan, Italy). Transwell^®^ insert was purchased from Millipore (Burlington, MA, USA). CellTiter 96^®^ AQueous One Solution Cell Proliferation Assay (MTS) was purchased from Promega (Madison, WI, USA). External standard for ICP-MS external calibration (IQC-026) was purchased from Agilent (Santa Clara, CA, USA), while internal standard (rodium) was purchased from Romil (Cambridge, Great Britain).

### 3.2. Formulations Composition

The comparative intestinal Fe absorption on an intestinal epithelium in vitro model was performed between six iron dietary supplements. Tested formulations were sorted based on their specific iron deficiency treatment, and their details are reported in Table 6. The commercial name of tested iron-based dietary supplements is reported in the Supplementary Materials (Table S1).

### 3.3. Methods

#### 3.3.1. Cell Cultures

##### Caco-2 Cell Culture

The human epithelial colorectal adenormacinoma Caco-2 cells (passage 30 to 40) were maintained in Caco-2 cell culture medium (Caco-2 CCM) (DMEM High Glucose medium supplemented with 10% FBS, 2% L-glutamine, 1% NEAA, and 1% Penicillin-Streptomycin mix). The cells were grown in a controlled atmosphere incubator (85% relative humidity, 5% CO_2_ and 37 °C). Caco-2 cells were seeded at 2000 cell/cm^2^ and medium changed every other day. Cells were subcultivated by tryspinization every 7 d when 80–90% confluent.

##### Raji Cell Culture

Human Burkitt’s lymphoma derived Raji cells (passage from 52 to 62) were maintained in Caco-2 CCM. Cells were cultured at a density of 1 × 10^5^ cells/mL in controlled atmosphere incubator (85% relative humidity, 5% CO_2_ and 37 °C) and subcultured twice a week, at a density of 1 × 10^5^ cell/mL.

#### 3.3.2. Iron Content Determination in the Different Iron-Based Formulations

The iron content of the different iron-based formulations was determined by inductively coupled plasma mass spectrometry. Briefly, a sample of iron supplement equivalent to 300 mg was mineralized with nitric acid (1 mL) in a thermoblock at 70 °C for 8 h. If this process was not sufficient to completely digest the sample, complete mineralization was achieved with a microwave-based digestion system (MARS2, CEM Corporation, Matthews, NC, USA), with a two-step heating ramp. During the first step, the temperature was raised to 140 °C in 15 min and reached temperature hold for 1 min, while in the second step, the temperature was increased to 180 °C in 15 min and the temperature was held for 10 min. Once mineralized, the digested iron supplement samples were transferred in pre-weighted polypropylene tubes, diluted up to 50 mL, and obtained solution were weighted. Further dilution was applied when necessary. Iron (*m*/*z* = 53) content was determined with a NexIon 300D (PerkinElmer, Waltham, MA, USA), using an external calibration and rodium as the internal standard. The measured amounts were compared to the declared ones and iron recovery was calculated as the percentage ratio between the measured and declared amount.

#### 3.3.3. Determination of Fe-Based Formulation Bioaccessibility

An amount corresponding to the daily intake suggested by each formulation posology (see Table 1) was exposed to an in vitro digestion procedure, which is designed to simulate the physiological process in humans (i.e., oral, gastric, and intestinal compartments). Briefly, all simulant digestive juices (i.e., saliva, gastric juice, duodenal juice, and bile) were prepared according to [34] and pre-heated to 37 °C. The digestion started by adding saliva (pH = 6.8 ± 0.1) to the formulation and incubating the obtained bolus at 37 °C for 5 min under constant head-over-heels agitation to simulate the chewing phase. Subsequently, gastric juice (pH = 1.3 ± 0.1) was added to the bolus and pH checked and, if necessary, adjusted to 2.5 ± 0.5 with. The resulting chime was further incubated at 37 °C for 2 h under constant head-over-heel agitation to simulate gastric peristaltic movements. In the next phase, duodenal juice (pH = 8.1 ± 0.1), bile (pH = 8.2 ± 0.1) and sodium bicarbonate solution were added. The pH of obtained chyle was set at 6.5 ± 0.5 and it was rotated head-over-heels for another 2 h at 37 °C. Digestive fluids volumetric ratio was strictly respected by adding saliva, gastric juice, duodenal juice, bile and sodium bicarbonate in the following ratio: 1:2:2:1:0.3. At the end of the digestive process, Fe concentration in the complete digests was measured and compared to the applied levels in order to estimate the overall recovery of the process. After a centrifugation step at 2750 *g* for 5 min, Fe was measured in the pellet (i.e., excreted fraction) and the supernatant, the latter corresponding to the bioaccessible fraction. Fe concentration was measured by ICP-MS, with the method previously described. For the mineralization step, 0.5 mL of liquid samples (complete and supernatant) and vacuum-dried pellets (solid samples) were digested with nitric acid. Fe-based formulation bioaccessibility was expressed as amount (μg) and percentage (%) compared to initial iron content.

#### 3.3.4. Determination of Delivery System Resistance to the Digestive Process

Dynamic Light Scattering (DLS) analysis (Zetasizer Nano S, Malvern, UK) of the different delivery systems was performed on ultrapure water (UPW)-resuspended formulations and the bioaccessible fractions (i.e., supernatant) of obtained digests. While SS, ME + VitC and ME + Folate UPW resuspensions and bioaccessible fractions were tested undiluted, PIC and ISPC samples were diluted 1/100 while LS samples were diluted 1/10. All dilutions were performed in UPW. A comparison between obtained results was performed to assess if the different carriers (liposomes, sucrosomes, microencapsulated iron, etc.) retained their particulate form at the end of the digestion process. The size distribution of the particles was evaluated as a signal intensity function only, since the particles refractive index was not available. The conversion of the signal intensity distribution into the particles volume or number distribution can cause error propagation, since it requires some unavailable parameters (e.g., the particles refractive index). Particle size in size distributions was expressed as hydrodynamic diameter (nm). Primary peak, d10, d50, and d90 (corresponding respectively to the percentage of particles (10, 50, and 90%) with a hydrodynamic diameter inferior to the reported value), expressed in nm, were reported as results.

#### 3.3.5. Determination of Fe-Based Formulations Functional Iron Fraction

To evaluate the iron functional value of tested Fe based-formulations, the exchangeable iron fraction of obtained supernatants was measured. Briefly, aliquots collected from the bioaccessible (i.e., supernatant) fraction of formulations’ digest were loaded in a dialysis membrane, which allowed only for ionic, unbound iron diffusion (6–8 kDa cut-off), and dialyzed against ultrapure water (ddH_2_O) for 24 h. At the end of the dialysis period, the dialyzing solution and the dialyzed supernatant were collected, and their Fe content was measured by ICP-MS. The iron content of the dialyzed supernatants and dialyzing solutions were expressed as µg.

#### 3.3.6. Intestinal Epithelium In Vitro Model

The intestinal and intracellular absorption of bioaccessible Fe fractions was determined using a human intestinal in vitro model based on a co-culture of Caco-2 and Raji cells, cultured as functional monolayers in Transwell^®^ inserts. Briefly, following the method described by Rieux et al., 2005 [35], Caco-2 cells (1.5 × 10^5^ cell/insert) were seeded in the apical compartment and left to mature to functional enterocytes for two weeks. Apical (0.5 mL) and basolateral (1.5 mL) Caco-2 CCM was refreshed every other day. Following enterocytes maturation, Raji cells were added to the basolateral compartment (6.7 × 10^4^ cell/mL). Then, the co-culture was left to mature for another week to allow for enterocyte to microfold cells (M cells) phenotype differentiation of a fraction of enterocyte population. Cell culture medium was refreshed only apically with the same frequency described before. Finally, prior to iron absorption experiment, Raji cells were removed, and the basolateral compartment extensively washed. The resulting functional human follicle-associated epithelia (FAE) is characterized by polarized cells with morphological and functional aspects typical of enterocytes (i.e., microvilli presence, tight junctions, and P-glycoprotein) and M cells (i.e., transcytosis process). Prior to Fe absorption experiments, cell monolayers trans-electrical resistance (TEER) was measured with an epithelial volt/ohm meter (Millicell^®^ ERS2, Millipore) equipped with a chopstick sensor. Only FAE endowed with a TEER value > 300 Ω/cm^2^ were considered for absorption experiments.

#### 3.3.7. Evaluation of Fe Intestinal Absorption

The bioaccessible fractions of the three formulations were added to the apical compartment (1 mL) of the in vitro intestinal epithelium model, while cell culture medium with 1% FBS was placed in the basolateral compartment (1.5 mL). Digestive fluids (DF), without iron dietary supplements or drug, were added to the apical epithelium as negative control. After 1 and 3 h of incubation in controlled atmosphere incubator (85% relative humidity, 5% CO_2_ and 37 °C), and under constant agitation (100 rpm) to simulate intestinal luminal shear stress, apical, basolateral, and cellular (FAE epithelium) fractions were collected. The Fe content of collected aliquots (apical, basolateral, and cellular fractions) was determined by ICP-MS analysis. As for bioaccessibility pellet samples, the cellular fraction was vacuum-dried before mineralization.

#### 3.3.8. Determination of Intracellular Ferritin

For intracellular ferritin determination, the protocol described by Scheers et al. 2014 [36] was followed. Briefly, after treatment with Fe-based formulations bioaccessible fraction, FAE monolayers were washed multiple times to remove non-absorbed Fe and left to incubate in modified Caco-2 CCM (1% FBS) for additional 23 (1 h exposure) and 21 h (3 h exposure) to allow for ferritin expression. At the incubation end, FAE-composing cells were detached by trypsinization, pelleted, and sonicated in lysis medium (0.1 Triton X-100 in ddH_2_O supplemented with proteases inhibitor cocktail). Finally, the ferritin content of the different cell lysates was determined by the ELISA (Enzyme-Linked ImmunoSorbent Assay) technique, using a commercially available kit. Ferritin levels were normalized for the total protein content of each sample, and results were expressed as fold-change compared to control (digestive fluid-treated FAE).

#### 3.3.9. Vitality and Barrier Integrity of the Intestinal Epithelium Model

At the end of Fe intestinal absorption experiments, the cell viability and barrier integrity of the FAE epithelium model were assessed. Cell viability was evaluated by using MTS assay, which is based on the reduction of MTS tetrazolium compound by viable cells to generate a colored formazan product that can be quantified by measuring the absorbance at 490 nm. Therefore, cell viability is directly proportional to absorbance. Vitality results were expressed as percentage (%) compared to digestive fluid-treated FAE. Barrier integrity was evaluated by measuring both monolayers trans-epithelial electrical resistance (TEER) and apparent permeability (Papp). The TEER trend was determined by measuring FAE monolayers trans-epithelial electrical resistance before (pre-treatment), soon after (post-treatment), and following 24 h recovery from treatment (24 h recovery). TEER results were expressed, for each condition, as percentage (%) compared to pre-treatment TEER values. Apparent permeability (Papp) was assessed by measuring Lucifer Yellow (LY) permeability. LY is a polar tracer used to investigate the paracellular permeability of a cell monolayer, since it is unable to pass through intact tight junctions. Briefly, 0.5 mL of 100 µg/mL LY in HBSS were added to the apical compartment of intestinal epithelium in vitro model, while 1.5 mL of HBSS were placed in the basolateral compartment. Following 1 h incubation in static condition and controlled atmosphere, the basolateral buffer was collected, and the permeated amount of LY was determined spectrofluorimetrically with a multiwell plate reader (428 nm excitation and 536 emission). The permeability coefficient (Papp, cm/min) was calculated with the following formula:

*Papp* = (Δ*CV*)/(Δ*tAC*_0_) (1)

where Δ*C*/Δ*t* is the flow of the molecule being transported across the monolayer during the incubation time (mM/min), *V* is the volume of the basolateral compartment (cm^3^), *A* is the area of the membrane (cm^2^), and *C_0_* is the initial concentration of the molecule in the apical compartment. Apparent permeability results were expressed as cm/min.

#### 3.3.10. Statistical Analysis

Results were statistically analyzed by *t*-test, using OriginLab software (OriginLab Corporation, Northampton, MA, US). Experiments were performed in triplicate and results were presented as average ± standard deviation. A *p* value of ≤0.05 was considered significant.

## 4. Conclusions

Six formulations for the treatment of anemia in different population targets were tested by using an integrated in vitro approach based on the coupling of digestive process with intestinal mucosa. The integrated approach permitted disclosing formulation behavior during digestion and the propensity of the different delivery systems in favoring iron release, iron absorption, and ferritin expression. Except for ISPC (i.e., iron-succinylated proteins complex) and LS (i.e., liposome) that are endowed with a good bioaccessibility, all the other delivery systems provided a limited bioaccessibility. Bioavailability data and ferritin expression of micronized ferric iron-based formulations—ME + VitC and ME + Folate—are characterized by a fast-intestinal absorption, while SS + VitC and ISPC reach their maximum absorption at longer time exposure. The microencapsulated formulations (ME + VitC and ME + Folate) showed the best performance for general-purpose and obstetrics and gynecology therapeutic target while, for the pediatric one, liposomes (LS) ensure an overall better intestinal iron absorption compared to the polydextrose-iron complex (PIC).

It is noteworthy that performances of IronOne products were obtained with the same or even less iron daily dose than the other formulations. This is relevant from a therapeutic point of view, since lowering the iron daily dose, while retaining optimal intestinal bioavailability, could mitigate or remove part of hurdles (i.e., bad taste, side effects, etc.), limiting the patient compliancy toward oral iron supplementation-based therapy.

## Figures and Tables

**Table 1:** Fe amount/dose in tested formulations, expressed as mg/dose. Results are expressed as mean ± standard deviation. ME: Microencapsulation; SS: Sucrosome; LS: Liposome; PIC: Polydextrose-iron complex; ISPC: Iron-succinylated proteins complex.

	Declared Amount/Dose (mg/dose)	Measured Amount (mg/dose)	Recovery (%)
ME with vitamin C (ME + VitC)	30	29.5 ± 2.5	98.3
SS with vitamin C (SS + VitC)	30	27.7 ± 0.8	92.3
LS	11.3	12.7 ± 0.1	112.9
PIC	30	27.7 ± 0.1	92.2
ME with folate (ME + Folate)	30	27.8 ± 0.1	92.5
ISPC	40	34.9 ± 0.1	87.0

**Table 2:** Fe content values in digests and their fractions—i.e., supernatants (bioaccessible fraction) and pellet (i.e., excreted fraction)—for the different iron dietary supplements. Results are expressed as mean ± standard deviation. ME: Microencapsulation; SS: Sucrosome; LS: Liposome; PIC: Polydextrose-iron complex; ISPC: Iron-succinylated proteins complex.

	Complete	Supernatant		Pellet	
Formulation	mg	mg	%	mg	%
**General-Purpose Iron Supplements**					
ME + VitC	28.35 ± 2.14	0.74 ± 0.12	2.43 ± 0.32	26.84 ± 2.52	94.71 ± 8.72
SS + VitC	27.75 ± 0.85	1.61 ± 0.02	5.78 ± 0.07	26.83 ± 0.20	96.70 ± 0.72
**Pediatric Iron Supplements**					
LS	11.8 ± 0.0	6.2 ± 0.3	53.0 ± 2.5	6.0 ± 0.1	50.8 ± 0.5
PIC	29.2 ± 2.2	0.1 ± 0.0	0.5 ± 0.0	21.7 ± 1.7	74.3 ± 5.9
**Obstetrics and Gynecology Iron Supplements**					
ME + Folate	27.76 ± 0.08	0.02 ± 0.00	0.07 ± 0.00	28.62 ± 0.41	103.11 ± 1.46
ISPC	34.85 ± 0.03	32.54 ± 1.73	96.55 ± 4.93	0.53 ± 0.19	1.82 ± 0.55

**Table 3:** Hydrodynamic diameters of formulations before (UPW, ultrapure water) and after in vitro digestion (Supernatant). Primary peak, d10, d50, and d90 (corresponding respectively to the percentage of particles (10, 50 and 90%) with a hydrodynamic diameter inferior to the reported value) are reported as hydrodynamic diameter (nm). Primary peak is reported as mean ± standard deviation. ME: Microencapsulation; SS: Sucrosome; LS: Liposome; PIC: Polydextrose-iron complex; ISPC: Iron-succinylated proteins complex.

Formulation	Sample	Primary Peak (nm)	d10 (nm)	d50 (nm)	d90 (nm)
General-Purpose Iron Supplements					
ME + VitC	UPW	396.1 ± 64.3	17.1	267.7	511.2
	Surnatant	396.1 ± 60.4	131.8	369.5	571.5
SS + VitC	UPW	91.3 ± 6.3	41.0	92.2	210.5
	Surnatant	122.4 ± 21.6	64.2	325.6	4447.2
**Pediatric Iron Supplements**					
LS	UPW	825.0 ± 115.9	627.9	892.7	4381.5
	Surnatant	458.7 ± 86.2	140.6	425.5	1013.0
PIC	UPW	50.8 ± 7.9	26.6	75.5	422.4
	Surnatant	122.4 ± 74.8	40.3	117.6	369.5
**Obstetrics and Gynecology Iron Supplements**					
ME + Folate	UPW	255.0 ± 5.4	133.1	206.4	347.3
	Surnatant	531.2 ± 88.1	186.5	511.2	1053.9
ISPC	UPW	190.1 ± 41.2	46.4	151.0	402.0
	Surnatant	68.1 ± 52.07	44.2	141.3	486.5

**Table 4:** Percentage iron recovery of tested formulations at the end of in vitro digestive process and determination of functional (retained iron) and ionic (released iron) iron fraction by dialysis. Results are reported as mean ± standard deviation. ME: Microencapsulation; SS: Sucrosome; LS: Liposome; PIC: Polydextrose-iron complex; ISPC: Iron-succinylated proteins complex.

	Recovery (%)	Functional Iron (%)	Released Iron (%)
**General-Purpose Iron Supplements**			
ME + VitC	83.27 ± 0.01	85.02 ± 0.01	14.98 ± 0.00
SS + VitC	97.93 ± 0.02	68.45 ± 0.02	31.55 ± 0.00
**Pediatric Iron Supplements**			
LS	99.16 ± 0.01	71.15 ± 0.01	28.85 ± 0.01
PIC	88.70 ± 0.01	89.41 ± 0.03	10.59 ± 0.01
**Obstetrics and Gynecology Iron Supplements**			
ME + Folate	100 ± 0.10	87.71 ± 0.06	12.20 ± 0.01
ISPC	85.49 ± 0.02	56.13 ± 0.01	43.87 ± 0.01

**Table 5:** Bioavailability (expressed as µg), ferritin expression (fold-change), and apparent permeability (Papp; cm/min) after 1 h and 3 h incubation for general-purpose, pediatric and obstetrics, and gynecology iron supplements. ME: Microencapsulation; SS: Sucrosome; LS: Liposome; PIC: Polydextrose-iron complex; ISPC: Iron-succinylated proteins complex.

Bioavailability				
Formulation	Time (h)	Fe (µg)	Ferritin (Fold Change)	P_app_ (×10^−4^ cm/min)
**General-Purpose Iron Supplements**				
ME + VitC	1	0.68 ± 0.04	18.3 ± 3.1	4.39 ± 0.18
	3	0.71 ± 0.06	17.8 ± 2.0	1.48 ± 0.06
SS + VitC	1	0.42 ± 0.13	12.8 ± 1.5	3.46 ± 1.65
	3	2.09 ± 0.64	24.2 ± 1.5	6.28 ± 2.23
**Pediatric Iron Supplements**				
LS	1	0.26 ± 0.08	17.1 ± 6.85	0.20 ± 0.04
	3	1.70 ± 0.12	12.4 ± 1.86	0.44 ± 0.04
PIC	1	0.00 ± 0.00	1.0 ± 0.2	0.00 ± 0.00
	3	0.00 ± 0.00	1.0 ± 0.1	0.00 ± 0.00
**Obstetrics and Gynecology Iron Supplements**				
ME + Folate	1	0.06 ± 0.02	0.3 ± 0.2	0.77 ± 0.25
	3	0.08 ± 0.01	1.4 ± 0.0	0.35 ± 0.05
ISPC	1	0.03 ± 0.02	4.3 ± 1.8	0.03 ± 0.02
	3	0.06 ± 0.04	6.4 ± 0.9	0.02 ± 0.01

**Table 6:** List of tested iron-based dietary supplement, sorted by therapeutic target.

Dietary Supplement	Total Fe Per Day (mg)	Iron	Delivery Technology	Other Active Principles
**General-Purpose Iron Supplements**				
ME + VitC	30.0	Fe^3+^ pyrophosphate	Microencapsulation	Vitamin C
SS + VitC	30.0	Fe^3+^ pyrophosphate	Sucrosomial Iron	Vitamin C
**Pediatric Iron Supplements**				
LS	11.3	Fe^2+^ fumarate	Liposomial Solution	-
PIC	30.0	Fe^3+^	Polydextrose-iron complex	-
**Obstetrics and Gynecology Iron Supplements**				
ME + Folate	30.0	Fe^3+^ pyrophosphate	Microencapsulation	Folate
ISPC	40.0	Fe^3+^	Iron-succinylated proteins complex	-

## Supplementary Materials

The following are available online, Figure S1: Dynamic Light Scatterig (DLS) size distribution of general-purpose iron supplements (ME + VitC (a) and SS + VitC (b)), pediatric iron supplements (LS (c) and PIC (d)) and obstetrics and gynecology iron supplements (ME + Folate (e) and ISPC (f)) in ultrapure water (UPW) and in bioaccessible fraction (Supernatant) of formulation digests, Table S1: Commercial name, codification and delivery systems of tested iron-based dietary supplement, Figure S2: Impact of iron supplements on trans-epithelial electrical resistance (TEER) of intestinal epithelia ((**a**): general-purpose iron supplements; (**b**): pediatric iron supplements; (**c**): obstetrics and gynecology iron supplements) following 1 h exposure.
