# Supplementary material for: Comparative Evaluation of Intestinal Absorption and Functional Value of Iron Dietary Supplements and Drug with Different Delivery Systems
Source: Molecules. 2020 Dec 17;25(24):5989. doi: 10.3390/molecules25245989 (PMC7766776; doi:10.3390/molecules25245989)
Supplement: Supplementary file 1 [file molecules-25-05989-s001.pdf]

Supplementary Materials

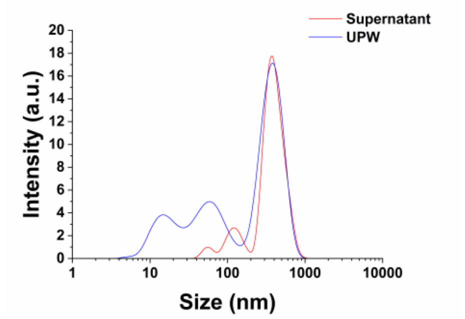

(a)

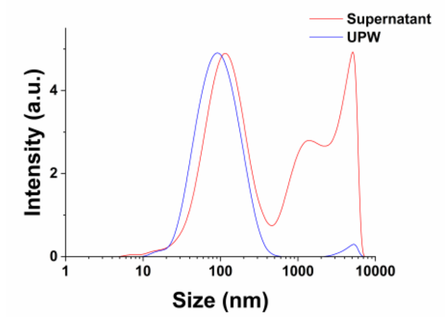

(b)

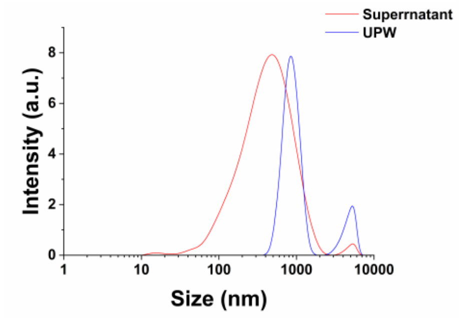

(c)

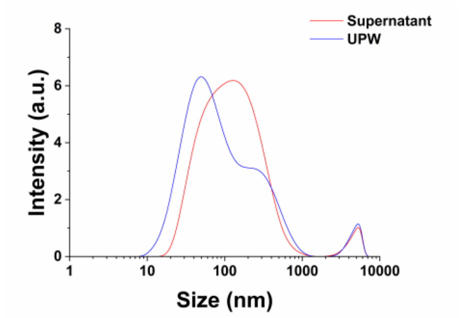

(d)

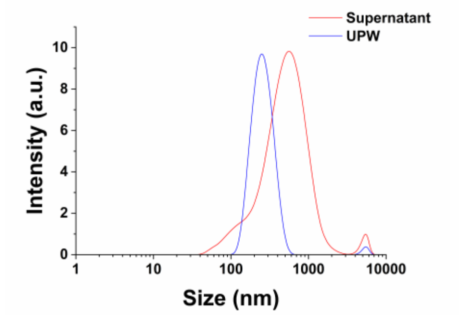

(e)

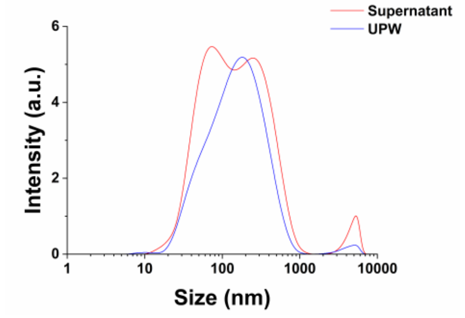

(f)

Figure S1. Dynamic Light Scatterig (DLS) size distribution of general-purpose iron supplements (ME + VitC (**a**) and SS + VitC (**b**)), pediatric iron supplements (LS (**c**) and PIC (**d**)) and obstetrics and gynecology iron supplements (ME + Folate (e) and ISPC (**f**)) in ultrapure water (UPW) and in bioaccessible fraction (Supernatant) of formulation digests. Size is reported as hydrodynamic diameter (nm). ME: Microencapsulation; SS: Sucrosome; LS: Liposome; PIC: Polydextrose–iron complex; ISPC: Iron-succinylated proteins complex.

Table S1. Commercial name, codification and delivery systems of tested iron-based dietary supplement.

Formulation	Typology	Code	Delivery Technology
IronOne Forte*	Dietary Supplement	ME + VitC	Microencapsulation
Commercial formulation 1	Dietary Supplement	SS + VitC	Sucrosomial Iron
IronOne Junior*	Dietary Supplement	LS	Liposomial Solution

Commercial formulation 2	Dietary Supplement	PIC	Polydextrose-iron complex
IronOne Folate*	Dietary Supplement	ME + Folate	Microencapsulation
Commercial formulation 3	Drug	ISPC	Iron-Succinylated proteins complex

* The use of commercial names of these products has been agreed with BMG PHARMA.

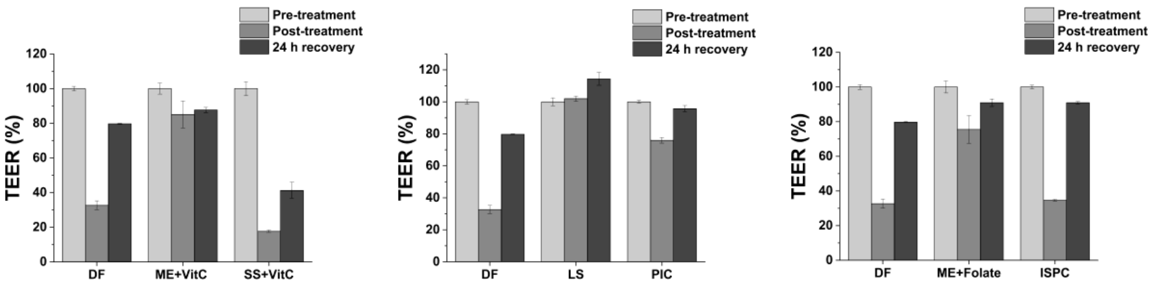

(**a**) (**b**) (**c**)

Figure S2. Impact of iron supplements on trans-epithelial electrical resistance (TEER) of intestinal epithelia (**a**: general-purpose iron supplements; **b**: pediatric iron supplements; **c**: obstetrics and gynecology iron supplements) following 1 h exposure. ME: Microencapsulation; SS: Sucrosome; LS: Liposome; PIC: Polydextrose–iron complex; ISPC: Iron-succinylated proteins complex; DF: Digestive fluids.
